# Bmi1-positive cells in the lingual epithelium could serve as cancer stem cells in tongue cancer

**DOI:** 10.1038/srep39386

**Published:** 2016-12-22

**Authors:** Toshihiro Tanaka, Naho Atsumi, Naohiro Nakamura, Hirotsugu Yanai, Yoshihiro Komai, Taichi Omachi, Kiyomichi Tanaka, Kazuhiko Ishigaki, Kazuho Saiga, Haruyuki Ohsugi, Yoko Tokuyama, Yuki Imahashi, Hiroko Hisha, Naoko Yoshida, Keiki Kumano, Kazuichi Okazaki, Hiroo Ueno

**Affiliations:** 1 Department of Stem Cell Pathology, Kansai Medical University, 2-5-1 Shin-machi, Hirakata, Osaka 573-1010, Japan; 2 Third Department of Internal Medicine, Kansai Medical University, 2-5-1 Shin-machi, Hirakata, Osaka 573-1010, Japan; 3 Department of Surgery, Kansai Medical University, 2-5-1 Shin-machi, Hirakata, Osaka 573-1010, Japan; 4 Department of Urology and Andrology, Kansai Medical University, 2-5-1 Shin-machi, Hirakata, Osaka 573-1010, Japan; 5 Department of Pediatrics, Kansai Medical University, 2-5-1 Shin-machi, Hirakata, Osaka 573-1010, Japan

## Abstract

We recently reported that the polycomb complex protein Bmi1 is a marker for lingual epithelial stem cells (LESCs), which are involved in the long-term maintenance of lingual epithelial tissue in the physiological state. However, the precise role of LESCs in generating tongue tumors and Bmi1-positive cell lineage dynamics in tongue cancers are unclear. Here, using a mouse model of chemically (4-nitroquinoline-1-oxide: 4-NQO) induced tongue cancer and the multicolor lineage tracing method, we found that each unit of the tumor was generated by a single cell and that the assembly of such cells formed a polyclonal tumor. Although many Bmi1-positive cells within the tongue cancer specimens failed to proliferate, some proliferated continuously and supplied tumor cells to the surrounding area. This process eventually led to the formation of areas derived from single cells after 1–3 months, as determined using the multicolor lineage tracing method, indicating that such cells could serve as cancer stem cells. These results indicate that LESCs could serve as the origin for tongue cancer and that cancer stem cells are present in tongue tumors.

Although lingual epithelial tissue is thought to be the origin of squamous cell carcinoma of the tongue, little is known about the cell types involved in tumorigenesis and whether cancer stem cells exist within the tumor. There are approximately 600,000 new cases of head and neck squamous cell carcinomas (HNSCCs) annually worldwide. HNSCCs usually develop in the oral cavity, oropharynx, larynx, or hypopharynx. Oral cancers are among the most common cancers, accounting for approximately 3% of all malignant tumors in both sexes^1^,^2^. Of these, tongue squamous cell carcinoma is highly aggressive, particularly when it occurs in young patients, and is often diagnosed in the advanced stages (stages III–IV), associated with a high metastasis rate and poor prognosis^3^,^4^. Because the 5-year survival rate has not improved substantially in the past 20 years for patients with tongue squamous cell carcinoma, it is important to elucidate the mechanism underlying tumorigenesis and tumor growth and to identify novel cancer stem cell markers for the development of new molecular-targeted therapies^5^.

Many studies have reported heterogeneity in the generation of human cancers and the existence of cancer stem cells that may explain resistance to radiological and chemical therapies^6^,^7^. For example, using mouse models, squamous cell carcinoma^8^ and pancreatic ductal carcinoma^9^ were shown to be heterogeneous. However, the strict verification of cancer stem cells *in vivo* is still necessary. We recently reported that Bmi1-positive cells are involved in the long-term maintenance of the lingual epithelium in the physiological state and quickly repair the lingual epithelium after irradiation-induced injury^10^,^11^. However, it is not known whether these cells serve as tongue cancer stem cells. In this study, we adopted the multicolor lineage tracing method to analyze the role of Bmi1-positive cells in a mouse model of chemically induced tongue cancer.

## Results

### Histological features of chemically induced tongue cancer

**Figure 1:**
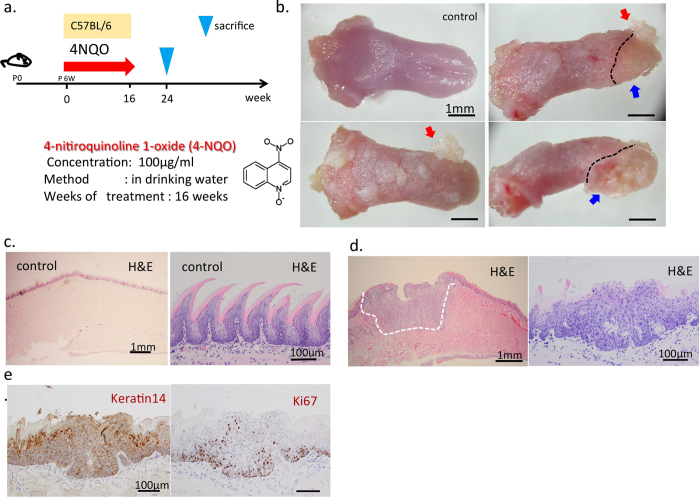
Morphological observations of tongue cancer and histological analysis of normal tongue and tongue cancer model. (**a**) Schematic representation of the timing of 4-NQO administration. (**b**) Morphological observations of normal tongues and tongues of cancer model mice. The red arrow indicates papilloma. The blue arrow indicates invasive carcinoma. (**c**) H&E staining of normal tongues and the filiform papillae (**d**) H&E staining of tongue cancer (**e**) Immunohistochemical staining of tongue cancer for Krt14 and Ki67. Scale bars: (**b**) 1 mm; (**c**) 1 mm (left), 100 μm (right); (**d**) 1 mm (left), 100 μm (right) (**e**) 100 μm.

4-NQO induces carcinomas in the oral cavities of mice^12^,^13^. In the current study, mice were administered 4-NQO (Fig. 1a) and more than 80% developed tongue cancers as well as esophageal cancers (Fig. 1b, Table 1). The tongues of 4-NQO-treated mice exhibited focal thickness and the lingual epithelium lacked organization (Fig. 1d), whereas the majority of the normal tongue epithelium was covered with aligned filiform papillae (Fig. 1c). We also observed both papillary or neoplastic squamous lesions (papillomas or carcinoma *in situ*) and invasive SCCs, identified by the invasion of neoplastic epithelial cells into the subepithelial tissues (Fig. 1d). Although cytokeratin-14 (Krt14) was specifically expressed in the basal layer of the normal lingual epithelium (Supplementary Fig. 1a), cancerous lesions lost this polarity, and Krt14 was uniformly expressed (Fig. 1e: left). In addition, the number of Ki67-positive cells was higher than that in the normal tongue epithelium (Fig. 1e: right, Supplementary Fig. 1b).

### Clonal analysis of the origin of tongue cancer

To determine if the origin of 4-NQO-induced tongue tumors is monoclonal, we labeled all epithelial cells with random colors and followed their fates in Rosa26^CreERT2/rbw^ mice^14^,^15^,^16^. Compared with conventional lineage tracing methods^17^, this system enables the clonal origin of tumors to be visualized and each cell cluster in the tumor derived from different clones could be easily distinguished based on color, as previously reported^11^,^16^,^18^,^19^,^20^. Using this system, we recently found that in the normal lingual epithelium, long-term stem cells are located in the interpapillary pit (IPP)^11^. The stem cells continuously self-renew and produce cells such that, at 4 weeks after labeling, the IPP area is occupied by single-colored cells, all of which are derived from a single stem cell. To determine the number of such cell clusters present in tongue tumors and to examine the clonal origin of these tumors, we labeled all cells of the tongue before inducing carcinogenesis by 4-NQO (Supplementary Fig. 2a). The IPPs in noncancerous tongue lesions in 4-NQO-treated mice showed focal thickness (hyperplasia) and were segmented into single-colored areas as well as normal lingual epithelia (Supplementary Fig. 2b). Both neoplastic squamous lesions (carcinoma *in situ*) (Supplementary Fig. 2c,d) and invasive SSCs (invasive carcinoma) (Supplementary Fig. 2e) were composed of several cell clusters. Taken together, these results suggest that each unit of the tumor was generated by a single cell, the assembly of which led to the formation of a polyclonal tumor.

### Presence of cancer stem cells in developing tongue cancers

**Figure 2:**
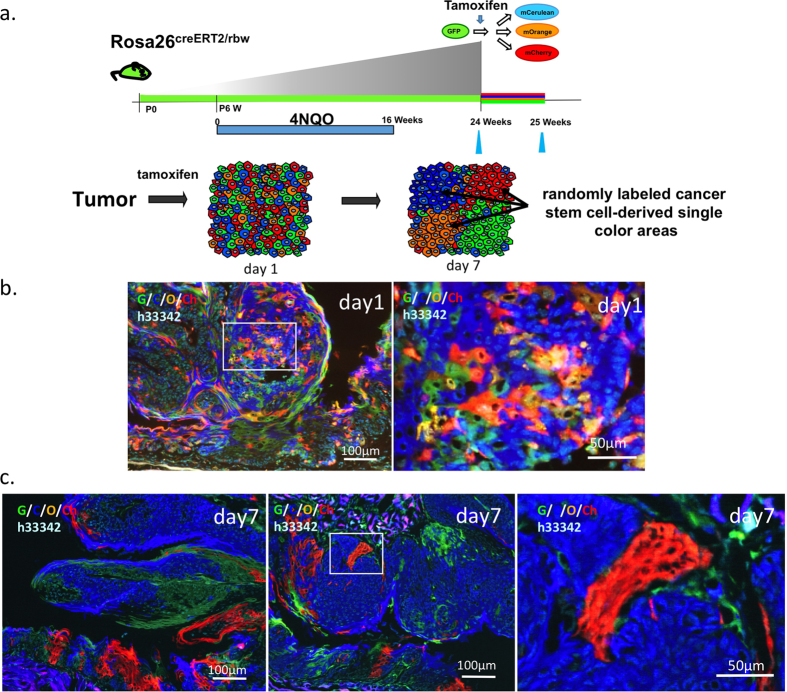
Rosa26^CreERT2/rbw^ mice were labeled with tamoxifen and induced by 4-NQO after the induction of tongue cancer. Schematic representation of the outcome of multi-color lineage tracing in the Rosa26^CreERT2/+^ tongue cancer model and timing of 4–NQO and tamoxifen treatment. (**b,c**) Rosa26^CreERT2/rbw^ mice were injected with tamoxifen after inducing tongue cancer and analyzed at the indicated time points (day 1 and day 7). Scale bars: (**b**) 50 μm (right), 100 μm (left); (**c**) 50 μm (right), 100 μm (left and middle).

We checked for the presence of cancer stem cells in developing tumors using the multicolor lineage tracing method. Carcinogenesis was induced in Rosa^creERT2/rbw^ mice, and tamoxifen injection was used to label all cells in the lingual epithelium (Fig. 2a,b). At 1 day after tamoxifen induction, the tumor cells were found to be randomly labeled (Fig. 2b). At day 7, several patches, each of which consisted of single-colored cells, were found in the tongue papilloma and carcinoma, indicating that certain cells in tongue cancer continuously proliferated and supplied tumor cells to the surrounding area (Fig. 2c). These results suggested that tongue cancer stem cells were present in the developing tumor.

### Bmi1-positive cells could serve as cancer stem cells

**Figure 3:**
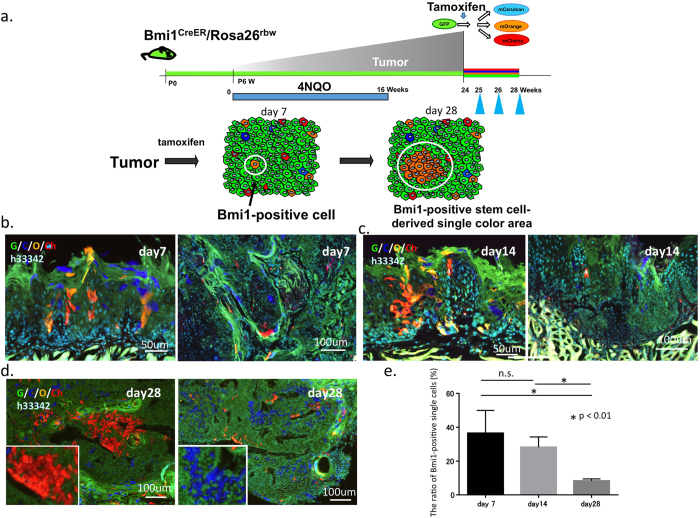
Fate of cells derived from Bmi1-positive cells in tongue cancer. (**a**) Schematic representation of the outcome of multi-color lineage tracing in the Bmi1^creER/+^/Rosa26^rbw/+^ tongue cancer model. (**b,c,d**) Bmi1^creER/+^/Rosa26^rbw/+^ mice were injected with tamoxifen and analyzed at the indicated time points (day 7, day 14, and day 28). (**e**) The ratio of single Bmi1-positive cells in the tumor (Bmi1^creER/+^/Rosa26^rbw/+^ mice, N = 6; day 7: n = 39 clones, day 14: n = 29 clones, day 28: n = 123 clones). *p < 0.01. Scale bars: (**b**) 50 μm (left), 100 μm (right); (**c**) 50 μm (left), 100 μm (right); (**d**) 100 μm.

To determine whether Bmi1-positive cells in tongue cancer serve as cancer stem cells, we used the gene-specific multicolor lineage tracing method to test if Bmi1-positive cells form patches in mice with tongue cancer as well as in Rosa^creERT2/rbw^ mice (Fig. 2c). To this end, after tumor induction by 4-NQO in Bmi1^creER/+^/Rosa26^rbw/+^ mice, the Bmi1-positive cells in the resultant tumors were labeled with tamoxifen and analyzed at various time points (Fig. 3a). The initial observation at day 7 after labeling revealed that Bmi1-positive cells were scattered in the developing tumors (Fig. 3b). Some of these cells then proliferated to form patches, which could be observed at 2–4 weeks after tamoxifen induction (Fig. 3c,d), demonstrating their continuous proliferation and supply of tumor cells. Although the tracing period was limited to 4 weeks owing to mouse death, the results indicate that at least some Bmi1-positive cells could serve as cancer stem cells, aiding the long-term maintenance of developing tumors. The number of Bmi1-positive cells that remained as single cells gradually decreased in the tongue tumors (Fig. 3e), suggesting that Bmi1 was also expressed in differentiated cells, which could neither self-renew nor supply tumor cells; the cells underwent terminal differentiation and finally disappeared.

## Discussion

To prevent carcinogenesis and treat cancer, it is extremely important to identify the origin of the cancer. In our study, carcinoma *in situ* or invasive SSC was composed of several cell clusters, each of which was derived from a different clone. By labeling Bmi1^+^ cells in Bmi1^creER/+^/Rosa26^rbw/+^ mice prior to inducing carcinogenesis, we examined whether tongue cancer originated from Bmi1^+^ LESCs. However, we could not detect single-colored tumors, i.e., monoclonal tumors, even 24 weeks after carcinogenesis induction (data not shown).

Although these results indicate that tongue cancer was polyclonal, they do not suggest a polyclonal origin. Rather, a better explanation for the observation that a single tumor was clearly segmented is that each unit of the tumor was generated from a single cell and multiple monoclonal tumors simultaneously developed and aggregated. This was probably because the method randomly induces multiple cancers and is therefore not appropriate for investigations of specific cells, such as Bmi1^+^ tongue stem cells, in tumor generation. We also analyzed Bmi1^CreERT/+^/Rosa26^lsl-KrasG12D/rbw^ mice in which the Kras^G12D^ mutation was induced in Bmi1-positive cells by tamoxifen, we could not detect any tumors in the tongue nor the oral mucosa. It may be useful to attempt to induce additional mutations, such as p53 or PTEN mutations.

We found that Bmi1^+^ cells produced clusters of single-colored cells in developing tumors, suggesting that Bmi1^+^ tumorigenic cells behaved as cancer stem cells and continually provided transit-amplifying cells in tongue tumors, contributing to tumor growth. In the same experiment, Bmi1^+^ cells that remained as single cells were also observed in the tumors at 28 days after labeling. One possibility is that they were differentiated cells, and could not proliferate further. Although immunostaining of rainbow colored-tumors to detect Ki67-positive cells might be helpful to distinguish fast-cycling cells, slow-cycling cells, and differentiated cells, this was unfortunately not possible owing to technical limitations. Co-staining of Ki67 is also helpful to distinguish between trans-amplifying cells and differentiated cells, but is not technically viable in this multicolor model. Another possibility is that they were resting cancer stem cells. In the physiological state, differentiated and labeled Bmi1^+^ cells in the lingual epithelium of Bmi1^creER/+^/Rosa26^rbw/+^ mice were terminally differentiated and disappeared within 7 days after labeling (data not shown). Furthermore, we did not observe Bmi1^+^ LESCs that remained in the resting state for more than 28 days. This could be attributed to the tumor environment surrounding these cells or their newly acquired characteristics after canceration. We could not determine which of these two possibilities was applicable to the resting Bmi1^+^ tumor cells, mainly because the mice with tongue tumors did not survive for a sufficiently long period.

The molecular mechanisms that underlie the regulation of tongue cancer stem cells by Bmi1 need to be investigated. It has been reported that Bmi1^+^ cells in pancreatic adenocarcinomas can self-regenerate^21^,^22^. Bmi1 is also associated with cancer stem cell markers, such as CD44 and Sox2^23^,^24^. Another study has also suggested that Bmi1 could serve as a molecular target for tongue cancer treatment, as Bmi1 overexpression is associated with invasion and poor prognosis in tongue squamous cell carcinoma^25^. Downregulation of Bmi1 with a small molecule, such as PTC 209 (*N*-(2,6-dibromo-4-methoxyphenyl)-4-(2-methylimidazo[1,2-a]pyrimidin-3-yl)-2-thiazolamine), inhibits the tumorigenic potential in colorectal cancer^26^. It is important to determine whether this molecule is also effective in tongue cancer.

Thus, we propose that at least some Bmi1^+^ tumorigenic cells behave as tongue cancer stem cells in developing tumors. Further studies are required to clarify the relationship between these cancer stem cells and Bmi1^+^ LESCs and to identify the cells of origin in tongue cancer.

## Methods

### Mice

Mice were bred and maintained at the Kansai Medical University Research Animal Facility in accordance with the Kansai Medical University guidelines. C57BL/6 J, Bmi1^CreER/+^^27^, Rosa26^CreERT2/+^, and Rosa26^rbw/+^ mice were purchased from Jackson Laboratories (Sacramento, CA, USA) or generated as previously described^15^,^16^,^17^. The experiments were approved by the Kansai Medical University Welfare Committee. Tamoxifen (Sigma, St. Louis, MO, USA) was dissolved in corn oil (Sigma) and intraperitoneally injected into adult mice at concentrations of 9 and 5 mg/40 g body weight for Bmi1^CreER/+^ mice and Rosa26^CreERT2/+^ mice, respectively.

### Chemically induced tongue tumors

4-Nitroquinoline-1-oxide (4-NQO) (Wako Pure Chemical Industries, Osaka, Japan) stock solution was diluted (final concentration, 100 μg/ml) in drinking water and administered to seven C57BL/6 mice, six Bmi1^creER/+^/Rosa26^rbw/+^ mice, and seven Rosa26^CreERT2/rbw^ mice. Starting from 16 weeks, the benign or malignant tumors that developed in the oral cavity and tongue were analyzed at various time points up to 28 weeks.

### Histological analyses

Mice were sacrificed, and the tissues were fixed, frozen, cut, and analyzed as previously reported^11^,^18^,^19^. Immunostaining was performed using the primary antibodies Ki67 (1:50; Dako, Glostrup, Denmark), cytokeratin 14 (Krt14, 1:2000; Covance, NJ, USA), and peroxidase- (1:200; Invitrogen, Carlsbad, CA, USA) as described previously^11^. Hematoxylin and eosin staining was performed using a standard protocol.

### Statistical analysis

The ratio of the single Bmi1-positive cells to the number of Bmi1-positive clusters was assessed in a section of the tongue tumor from six Bmi1^creER/+^/Rosa26^rbw/+^ mice in which tumorigenesis was induced by 4-NQO. Day 7: n = 39 clones, mean 36.48, s.d. 13.46; day 14: n = 29 clones, mean 28.18, s.d. 6.17; day 28: n = 123 clones, mean 8.33, s.d. 1.15.

### Ethics statement

All animal experiments were performed in accordance with the Kansai Medical University guidelines and were approved by the Kansai Medical University Animal Experiment Committee.

## Additional Information

**How to cite this article:** Tanaka, T. *et al*. Bmi1-positive cells in the lingual epithelium could serve as cancer stem cells in tongue cancer. *Sci. Rep.* **6**, 39386; doi: 10.1038/srep39386 (2016).

**Publisher's note:** Springer Nature remains neutral with regard to jurisdictional claims in published maps and institutional affiliations.

## Supplementary Material

Supplementary Information

## Figures and Tables

**Table 1:** 4-NQO induction of tongue cancer in C57BL/6, Bmi1^creER/+^/Rosa26^rbw/+^ mice, and Rosa26^CreERT2/rbw^ mice.

Mouse strain	Method	No. of mice at 0 weeks	No. of surviving mice at 24 weeks	4-NQO concentration	Weeks of treatment	Incidence of papilloma	Incidence of invasive SSC	Incidence of esophageal SCC
**C57BL/6**	**Control**	3	3	**0 μg/ml**	16	0	0	0
**C57BL/6**	**4NQO**	7	5	**100 μg/ml**	16	2	3	5
**Bmi1**^**CreER/+**^**/Rosa26**^**rbw/+**^	**4NQO**	6	6	**100 μg/ml**	16	4	6	6
**Rosa26**^**CreERT2/rbw**^	**4NQO**	7	5	**100 μg/ml**	16	3	5	4

Mice were treated with 4-NQO in drinking water for 16 weeks and then observed for another 8 weeks. We investigated the tongue and esophagus of mice at the indicated time points.
