# Supplementary material for: Bmi1-positive cells in the lingual epithelium could serve as cancer stem cells in tongue cancer
Source: Sci Rep. 2016 Dec 22;6:39386. doi: 10.1038/srep39386 (PMC5177893; doi:10.1038/srep39386)
Supplement: Supplementary Information [file srep39386-s1.pdf]

Bmi1-positive cells in the lingual epithelium could serve as cancer stem cells in tongue cancer

[1,2,3]Toshihiro Tanaka, [1]Naho Atsumi, [1,3]Naohiro Nakamura, [1,4]Hirotsugu Yanai, [1,5]Yoshihiro Komai, [1,6]Taichi Omachi, [1]Kiyomichi Tanaka, [1]Kazuhiko Ishigaki, [1]Kazuho Saiga, [1,5]Haruyuki Ohsugi, [1]Yoko Tokuyama, [1]Yuki Imahashi, [1]Hiroko Hisha, [1]Naoko Yoshida, [1]Keiki Kumano, [2]Kazuichi Okazaki and [1]Hiroo Ueno*

[1]Department of Stem Cell Pathology, [2] Research Fellow of Japan Society for the Promotion of Science,, [3]Third Department of Internal Medicine, [4]Department of Surgery, [5]Department of Urology and Andrology and [6]Department of Pediatrics, Kansai Medical University, 2-5-1 Shin-machi, Hirakata, Osaka 573-1010, Japan

*Correspondence and requests for materials should be addressed to H.U. (hueno@hirakata.kmu.ac.jp).

LESC: lingual epithelial stem cell
HNSCC: head and neck squamous cell carcinoma
TSCC: tongue squamous cell carcinoma
SSC: squamous cell carcinoma
4NQO: 4-nitroquinoline-1-oxide
Krt14: cytokeratin 14
IPP: intrapapillary pit

Supplementary Figure

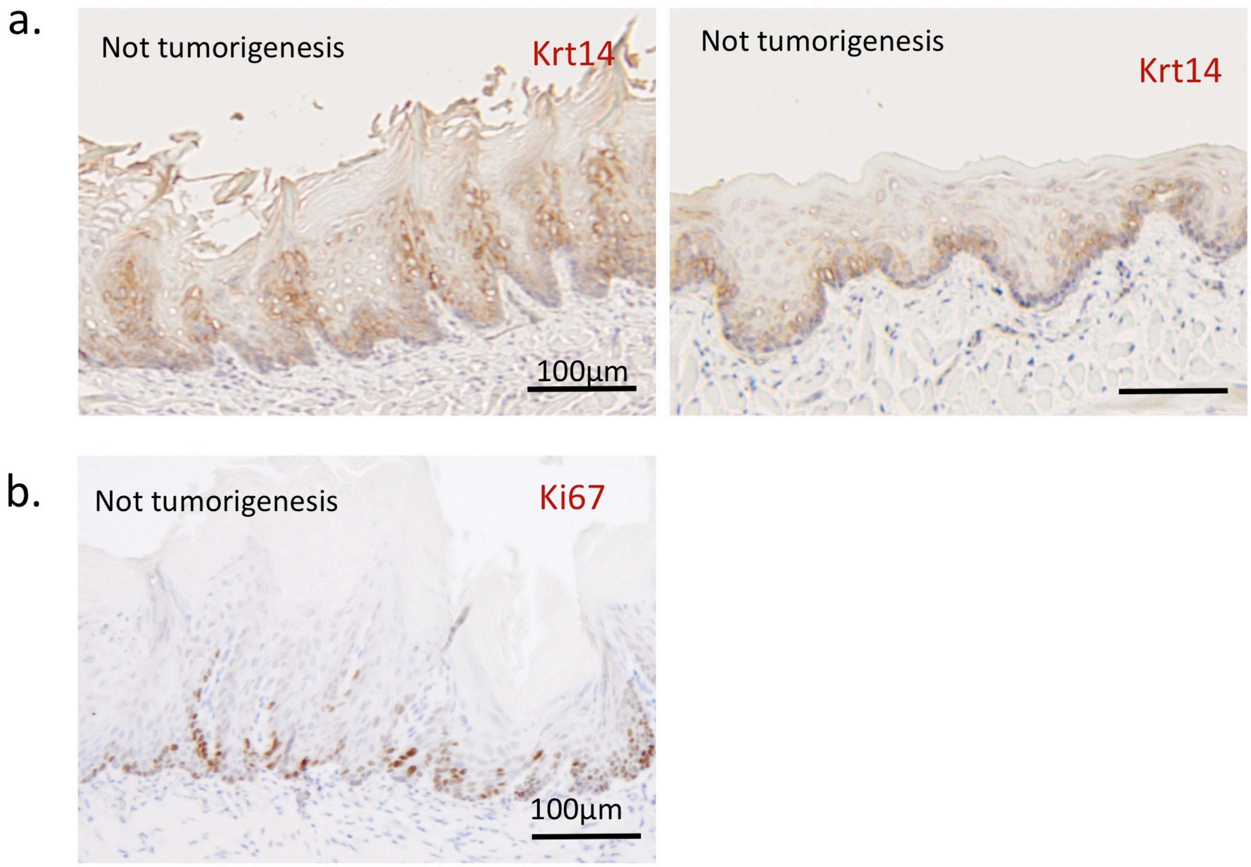

Supplementary Fig 1　Immunostaining of the tongue epithelium

a, b. Immunostaining of tongue epithelium with Krt14 (a) and Ki67 (b). Scale bar: a. b.:100µm

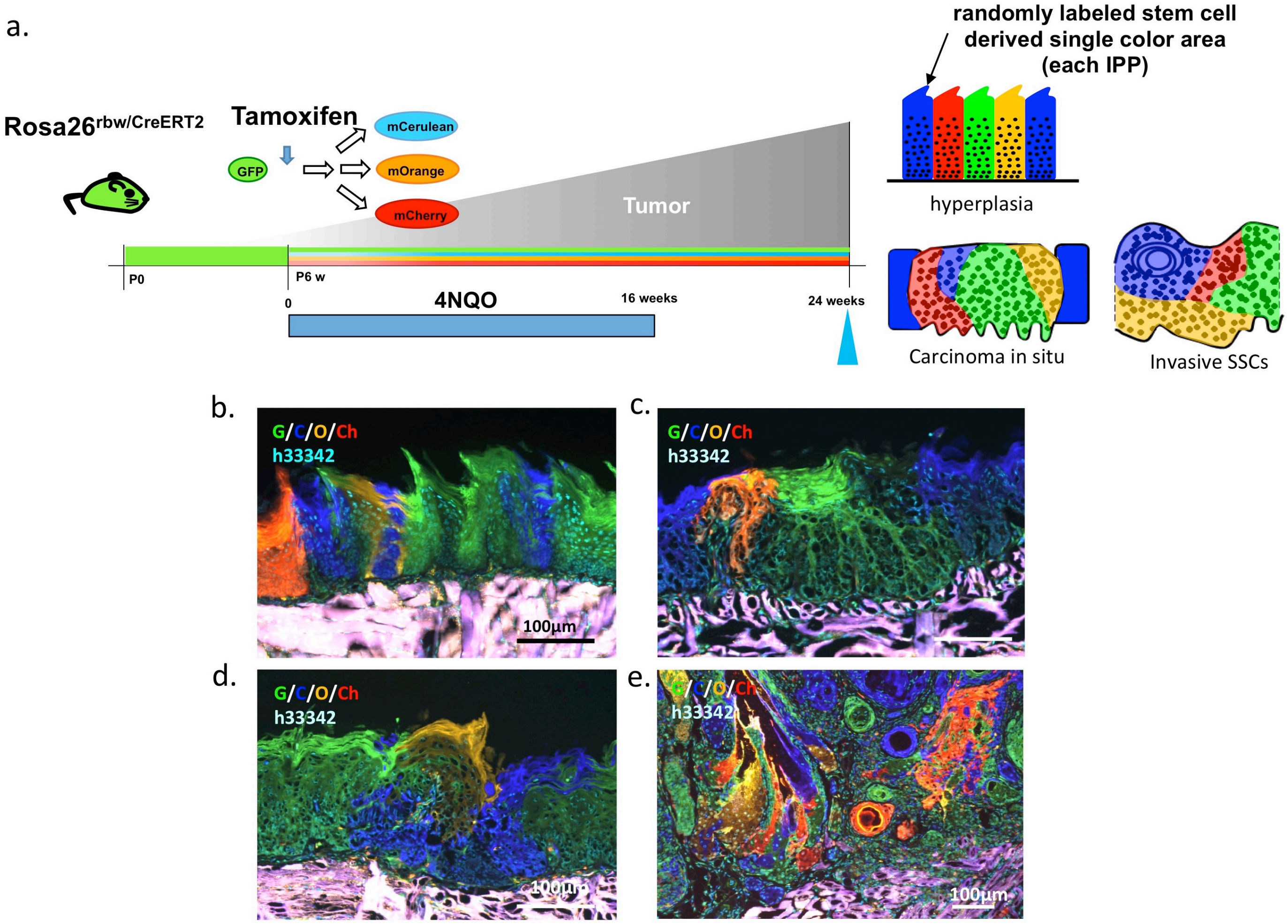

Supplementary Fig 2 Analysis of clonality of tongue cancer in Rosa26$^{CreERT2/rbw}$ mice

a. Schematic representation of the timing of tamoxifen and 4-NQO administration. Schematic representation of tissue maintenance with Rosa26$^{CreERT2/rbw}$ mice in physiological condition, hyperplasia and carcinoma. b. Rosa26$^{CreERT2/rbw}$ mice were labeled with tamoxifen and induced by 4-NQO (hyperplasia). c.d.e. Rosa26$^{CreERT2/rbw}$ mice were labeled with tamoxifen and induced by 4-NQO (carcinoma in situ and invasive carcinoma). Scale bar: b, c, d, e :100μm
